# Increased Expression on Innate Immune Factors in Placentas From HIV-Infected Mothers Concurs With Dampened Systemic Immune Activation

**DOI:** 10.3389/fimmu.2020.01822

**Published:** 2020-08-25

**Authors:** Nátalli Zanete Pereira, Anna Cláudia Calvielli Castelo Branco, Kelly Cristina Gomes Manfrere, Josenilson Feitosa de Lima, Fabio Seiti Yamada Yoshikawa, Helaine Maria Besteti Pires Mayer Milanez, Naiura Vieira Pereira, Miriam Nacagami Sotto, Alberto José da Silva Duarte, Maria Notomi Sato

**Affiliations:** ^1^Laboratório de Investigação em Dermatologia e Imunodeficiências LIM56, Instituto de Medicina Tropical, Faculdade de Medicina FMUSP, Universidade de São Paulo, São Paulo, Brazil; ^2^Departamento de Dermatologia, Faculdade de Medicina FMUSP, Universidade de São Paulo, São Paulo, Brazil; ^3^Instituto de Ciências Biomédicas - Universidade de São Paulo, São Paulo, Brazil; ^4^Division of Molecular Immunology, Medical Mycology Research Center, Chiba University, Chiba, Japan; ^5^Departamento de Tocoginecologia, Universidade Estadual de Campinas, Campinas, Brazil

**Keywords:** DAMPs, inflammation, cord blood, HIV-infection, placental tissue, newborns

## Abstract

Innate immunity is one of the main protection mechanisms against viral infections, but how this system works at the maternal-fetal interface, especially during HIV infection, is still poorly known. In this study, we investigated the relationship between pregnancy and innate mechanisms associated with HIV immunity by evaluating the expression of DAMPs, inflammasome components and type I/III IFNs in placenta and serum samples from HIV-infected mothers and exposed newborns. Our results showed that most of these factors, including HMGB1, IL-1, and IFN, were increased in placental villi from HIV-infected mothers. Curiously, however, these factors were simultaneously repressed in serum from HIV-infected mothers and their exposed newborns, suggesting that pregnancy could restrict HIV immune activation systemically but preserve the immune response at the placental level. An effective local antiviral status associated with a suppressed inflammatory environment can balance the maternal immune response, promoting homeostasis for fetal development and protection against HIV infection in neonates.

## Introduction

Human immunodeficiency virus (HIV) infection and acquired immunodeficiency syndrome (AIDS) are public health problems with high morbidity and mortality rates, with 36.9 million people worldwide estimated to be infected (1). Albeit in maternal HIV infection, infected cells and virions pass through the placenta, either free or associated with neutralizing antibodies, most of HIV-exposed children do not become infected, even without any antiretroviral therapy (ART). Nonetheless, these children show a remarkable immune impairment throughout their lives, highlighting the need to better understand the mechanisms involved in HIV infection during pregnancy (2).

Despite the well-known effects of HIV over the adaptive immune system, the innate immunity has been recognized as an important player in host defense but also in disease pathogenesis. The classical innate antiviral system of type I interferons (IFN) is considered a potent inhibitor of HIV infection in CCR5+ CD4+ T cells and macrophages (3). Type I IFN promotes the positive regulation of several antiviral restriction factors, such as APOBEC (Apolipoprotein), TRIM (tripartite motif), and SAMHD1 (Sterile alpha motif and HD-domain containing protein 1) (4). Previously, we showed an upregulation of several antiviral factors in HIV-exposed newborns (5). Curiously, in addition to its defense role, the IFN response is essential for a successful gestation (6). Type III IFN (IFN-λ), for example, is constitutively expressed by trophoblasts and it was shown to inhibit replication of a number of viruses such as dengue virus, respiratory syncytial virus, herpes simplex virus, Zika virus (7–10), and HIV (11). Moreover, IFN-λ is enhanced in mouth epithelial cells from HIV-exposed adults (12). However, we still lack a deep *in situ* understanding of the IFN response in the placental niche during HIV infection.

Other innate systems were also linked to HIV pathogenesis. Expression and activation of inflammasome components, for example, as caspase-1, IL-1β, and IL-18, were linked to viral replication (13). But endogenous molecules known as alarmins or DAMPs (damage associated molecular patterns), responsible for signaling damage to cells and tissues (14), could help in viral restriction, as exemplified by the ability of HMGB1 (high-mobility group proteins) in inhibiting HIV-1 replication in primary macrophages by inducing the release of chemokines that compete for viral entry receptors (15). Whether and how these networks are involved in HIV infection during pregnancy remains to be uncovered.

In this study, we investigated the balance between inflammatory and antiviral factors in placentas of HIV-infected mothers. Even though HIV infection is known to promote a state of chronic immune activation, we observed that infected mothers and exposed newborns present a diminished inflammatory response systemically. Strikingly, their placentas presented an opposite profile, with heightened expression of DAMPs, inflammasome components, and IFNs, which could help to restrict vertical transmission.

## Methods

### Ethics

This study was approved by the Institutional Use Committee of the University of São Paulo and University of Campinas (Plataforma Brasil database, CAAE: 31605314.3.0000.0068). All mothers provided written consent on the behalf of their newborn participants.

### Study Sample

Twenty-six HIV-infected mothers were recruited from obstetrics outpatient clinics at the “Hospital das Clínicas” of the University of São Paulo (HC/FMUSP) and the “Centro de Atendimento Integral a Saúde da Mulher (CAISM)” of the University of Campinas. All infected mothers underwent an elective cesarean section, were undergoing ART, and had received prophylactic intravenous zidovudine 3–5 h prior to cesarean section. As a control group, 31 HIV-uninfected (UN) pregnant women undergoing cesarean section (iterativity) and 2 HIV-uninfected (UN) pregnant women who had vaginal delivery were enrolled from the Section of Obstetrics from the “Hospital Universitário” of the University of São Paulo. All mothers were older than 18 years (18–36 years), presented term pregnancy, and had negative serology for hepatitis B and C viruses, syphilis, rubella, and toxoplasmosis. The main demographic characteristics are presented in Table 1.

**Table 1:** HIV-infected mothers profile.

**ID**	**WG (w)**	**ART**	**CD4 mm^**3**^**	**CD8 mm^**3**^**	**Ratio CD4/CD8**	**VL copies/mL**	**DT (y)**	**NBW (g)**	**NBS**	**PW (g)**	**P**	**D**	**A**
1	37.9	3TC/AZT + LPV/RTV	793	566	1.40	<50	–	2,500	F	330	1	0	0
2	37.7	TDF + ATV + RTV + 3TC/AZT	345	760	0.45	<50	12	2,750	F	–	4	3	0
3	38.1	3TC/AZT + RTV + LPV/RTV	156	745	0.21	<50	13	3,260	F	440	4	3	1
4	37.4	TDF + ATV + LPV/RTV	522	498	1.05	<50	18	2,380	M	590	1	0	0
5	37.8	3TC/AZT + LPV/RTV	540	713	0.76	<50	2	2,880	M	–	8	3	4
6	–	–	243	310	0.78	<50	–	–	–	–	–	–	–
7	38.3	AZT + LPV/RTV	392	662	0.59	<50	3	3,070	F	740	2	1	0
8	37.2	3TC/AZT + LPV/RTV	688	1,053	0.65	<50	5	3,430	F	670	3	2	1
9	38.4	3TC/AZT + NVP	666	–	–	1,911	5	3,420	M	–	4	0	3
10	37.3	3TC/AZT + LPV/RTV	856	725	1.18	<50	0	3,130	M	490	2	0	1
11	–	3TC/AZT + LPV/RTV	1,132	721	1.57	<50	–	2,820	M	–	2	1	0
12	–	3TC/AZT + LPV/RTV	517	474	1.09	<50	–	2,950	F	–	3	2	0
13	37.4	LPV/RTV + 3TC + RAL + TDF	327	734	0.45	<50	19	2,600	M	560	1	0	0
14	–	ABC + 3TC + LPV/RTV	828	2,035	0.41	<50	16	2,830	F	390	1	0	0
15	37.9	3TC/AZT + NVP	656	1,721	0.38	<50	1	2,900	F	500	3	1	1
16	37.6	3TC/AZT + LPV/RTV + TDF	243	310	0.78	<50	22	2,480	M	350	1	0	0
17	36.0	3TC/AZT + NVP	–	–	–	–	7	2,294	F	810	3	0	2
18	37.7	3TC/AZT + LPV/RTV	588	–	–	<50	0	2,400	F	360	1	0	0
19	37.7	AZT + 3TC + TDF	394	798	0.49	<50	13	2,850	F	500	5	2	2
20	38.2	3TC/AZT + LPV/RTV + TDF	842	625	1.35	<50	16	3,060	M	650	1	0	0
21	37.3	TDF + 3TC/AZT + RAL + DRV+ RTV	459	820	0.56	<50	18	2,720	F	480	1	0	0
22	37.7	3TC/AZT + LPV/RTV	326	–	–	<50	0	3,120	F	760	3	2	0
23	–	3TC/AZT + NVP	388	1,246	0.31	<50	–	1,960	M	250	1	0	0
24	39.1	ABC+3TC+ATV+RTV	1,003	–	–	<50	20	2,580	F	400	1	0	0
25	38.6	TNF+3TC+RAL	241	–	–	383	1	3,130	M	465	1	0	0
26	39	TNF+3TC+NVP	1,047	–	–	<50	–	3,695	M	665	3	1	1

*ID, identification number; WG, week of gestation at delivery; ART, antiretroviral therapy; VL, viral load; DT, diagnostic time; NBW, Newborn weight; NBS, Newborn sex; F, female; M, male; PW, Placenta weight; P, pregnancy; D, delivery; A, abortion; Y, years; g, grams; ART: 3TC, Lamivudine; TDF, Tenofovir; AZT, zidovudine; NVP, Nevirapine; RAL, Raltegravir; DRV, Darunavir; RTV, Ritonavir; LPV, Lopinavir; ABC, Abacavir; ATV, Atazanavir*.

### Isolation of Placental Tissues, Explants, and Serum Samples

Cotyledons (placental tissue) measuring 1 cm^2^ were cut from a random central area soon after the placenta was withdrawn and transported in saline, formaldehyde solution, or DMEM/F12 (Gibco, CA, USA). Samples were washed with saline solution for removal of residual blood and a section of 30 mg was obtained from decidua (maternal face) and villus (fetal face). Approximately 100 mg of villus fragments were grown in 24-well plates (Corning-Costar) containing DMEM / F12, 10% fetal bovine serum (SFB Gibco, CA, USA), 10 mg/ml gentamicin, 100 mg/ml penicillin/streptomycin, 1 mg/mL amphotericin B, 520 μg/mL sodium lactate, and 56 μg/mL sodium pyruvate at 37°C with 5% CO_2_, for 24 h. For polymerase chain reaction (PCR) assays, samples were conditioned in RNAlater solution (Sigma, St Louis, MO, USA) and frozen at −20°C for further analysis.

Serum samples were collected from peripheral blood post-delivery (mothers) or cord blood samples and stored at −70°C.

### Inflammation Induction

After resting, the placental explants were washed twice with DMEM/F12 and then stimulated with a TLR4 agonist [lipopolysaccharide (LPS) from *Salmonella enterica* serotype minnesota, Sigma-Aldrich St Louis, MO, USA - 1 μg/mL] for 24 h at 37°C with 5% CO_2_. After this period, cell supernatant was collected and stored at −70°C for soluble factors measurement.

### RT qPCR

Total RNA was extracted from stored placental tissues (maternal and fetal sides separately) using the commercial RNeasy Plus Mini Kit (Qiagen, Valencia, CA, USA). Reverse transcription was performed with an iSCRIPT Reverse Transcriptase Kit (Biorad, CA, USA). The oligonucleotides are detailed in Table 2. PCR reaction was performed in an Applied Biosystems 7500 system using specific oligonucleotides and SYBR Green (Applied Biosystems, Carlsbad, CA, USA) fluorescence detection reagents. The cycling protocol consisted of 10 min at 95°C, followed by 40 cycles of 15 s at 95°C and 60 s at 60°C. Amplification results were visualized and analyzed using Sequence Detection System (SDS) software (Applied Biosystems). Normalized expression was calculated as previously described and *GAPDH* was employed as internal control (16).

**Table 2:** List of oligonucleotides.

**Target**	**Sequence Forward 5^**′**^-3^**′**^**	**Sequence Reverse 5^**′**^-3^**′**^**
Pró-IL1-β (NM_000576.2)	TCCCCAGCCCTTTTGTTGA	TTAGAACCAAATGTGGCCGTG
NLRP1 (NM_033004.4)	AAGACCAGCTGTTCTCGGAGTT	AGGCATGAGATCTCCTGGTTTC
NLRP3 (NM_004895.4)	TGGAGTGTCGGAGAAGAG	TGCTGTCATTGTCCTGGTGT
Pró-IL-18 (NM_001562.4)	GACGCATGCCCTCAATCC	CTAGAGCGCAATGGTGCAATC
IRF3 (NM_0015171.6)	AGAGGCTCGTGATGGTCAAGGTT	AGAGTGGGTGGCTGTTGGAAATG
IFN-α (NM_024013.2)	AAATACAGCCCTTGTGCCTGG	GGTGAGCTGGCATACGAATCA
IFN-λ (NM_172140.2)	CGCCTTGGAAGAGTCACTCA	GAAGCCTCAGGTCCCAATTC
S100A8 (NM_001319196.1)	GGGATGACCTGAAGAAATTGCTA	TGTTGATATCCAACTCTTTGAACCA
S100A9 (NM_002965.3)	GTGCGAAAAGATCTGCAAAATTT	GGTCCTCCATGATGTGTTCTATGA
RAGE (NM_001136.4)	GAGGAGGAGCGTGCAGAACT	CCTCAAGGCCCTCCAGTACTACT
TLR4 (NM_138554.5)	CAGAGTTTCCTGCAATGGATCA	GCTTATCTGAAGGTGTTGCACA
TLR9 (NM_017422.3)	AAGGCCAGGTAATTGTCACGG	ACAACAACATCCACAGGCAAGT
HMGB1 (NM_001313893.1)	CTCAGAGAGGTGGAAGACCATGT	GGGATGTAGGTTTTCATTTCTCTTTC
IRF-7 (NM_001572.3)	TGGTCCTGGTGAAGCTGGAA	GATGTCGTCATAGAGGCTGTTG
IFI16 (NM_001206567.1)	AACCACGTTGAAACCAAGACT	TGCGTTCAGCACCATCACTT
AIM2 (NM_004833.2)	CACCAAAAGTCTCTCCTCATGTT	AAACCCTTCTCTGATAGATTCCTG
Caspase1 (NM_033292.3)	TTACAGACAAGGGTGCTGAACAA	TGAGGAGCTGGAAAGGAAGAAAG
ASC (NM_013258.4)	AAGCCAGGCCTGCACTTTAT	CTGGTACTGCTCATCCGTCA
B7-H3 (NM_001024736.2)	GGAGGAGAATGCAGGAGCTG	TGGTCCTCATGGTCAGGCTA
HLA-G (NM_001363667.1)	ATGCTGAGATGGAAGCAGTCTT	AGTCACAAAGGGACTTGCCA
GAPDH (NM_002046.6)	GAAGGTGAAGGTCGGAGT	GAAGATGGTGATGGGATTTC

### Placental Morphological Analysis and Immunohistochemistry

Placental tissues were formalin-fixed, embedded in paraffin, and sectioned at 4 μm-thick slices. Staining reactions were performed on silanized slides (Sigma Chemical Co., St. Louis, MO/USA). Slides were dewaxed in xylol baths, subsequently hydrated in decreasing concentrations of ethanol, and stained with hematoxylin-eosin and analyzed under a microscope.

For immunohistochemistry analysis, histological sections were deparaffinized in xylol baths and rehydrated in ethanol. Following blockade with 3% hydrogen peroxide, slides were incubated with primary antibodies anti-HMGB1, anti-IL-1β, anti-IFN-β, anti-STING, anti-NLRP3, anti-NLRP1, anti-AIM-2, anti-IL-18, anti-caspase-1, and anti-HLA-G (Abcam, Cambridge, MA, USA) diluted in phosphate buffered saline (PBS, pH 7.4) supplemented with 1% of bovine serum albumin (Sigma-Aldrich, St. Louis, MO). Reactions were visualized with Permanent Red LSAB-AP chromogenic solution (Dako, CA, USA) and detected in a Novolink Max Polymer Detection System (RE7280-K, Leica Biosystems, Newcastle Upon Tine, UK). Non-immune IgG was used as negative staining control. The slides were scanned using an Aperio Scan-scope Cs Scan (Aperio Technologies, Vista, CA) and analyzed in the Image-Pro Plus software (Media Cybernetics Inc., Bethesda, MA, USA). Total tissue distribution of proteins in the stained area divided by the total area measured in the decidua and villi was calculated for analysis.

### Cytokines/Chemokines and Alarmin Measurements

Serum samples were assessed for IL-6, IL-10, IL-4, IL-1β levels, and supernatant were assessed for IL-10, TGF-β, IL-6, TNF, and IL-1β levels by cytometric bead array (CBA) assay using commercial Cytokine Kit reagents (Becton Dickinson, San Jose, CA, USA). Data were acquired in BD LSRFortessa™ Cell Analyzer (BD Biosciences, CA, USA) and analyzed in the FCAP Array Software v3.0 (BD Biosciences, CA, USA).

Determination of HMGB1 was assessed by ELISA according to the manufacturer's instructions (BioLegend, San Diego, CA, USA).

The detection limits for serum analyzes were: IL-10 = 13.7 fg/mL; IL-6 = 68.4 fg/mL; IL-1β = 48.4 fg/mL; IL-4 = 144.4 fg/mL, and HMGB1 = 2.5 ng/mL. The detection limits for supernatant analyzes were: IL-10 = 0.13 pg/mL; IL-1β = 2.3 fg/mL; TGF-β = 14.9 pg/mL, and TNF = 0.7 pg/mL.

### Statistical Analysis

The Mann-Whitney *U* test was used to compare variables between HIV-infected mothers and healthy controls, and Wilcoxon signed-rank test was used for comparisons between paired samples, as maternal and CB samples. A *p-*value < 0.05 was considered significant.

## Results

### Enhanced Expression of DAMPs in Placental Tissue Parallels With Decreased HMGB1 Serum Levels in HIV-Infected Mothers and Their Exposed Newborns

**Figure 1:**
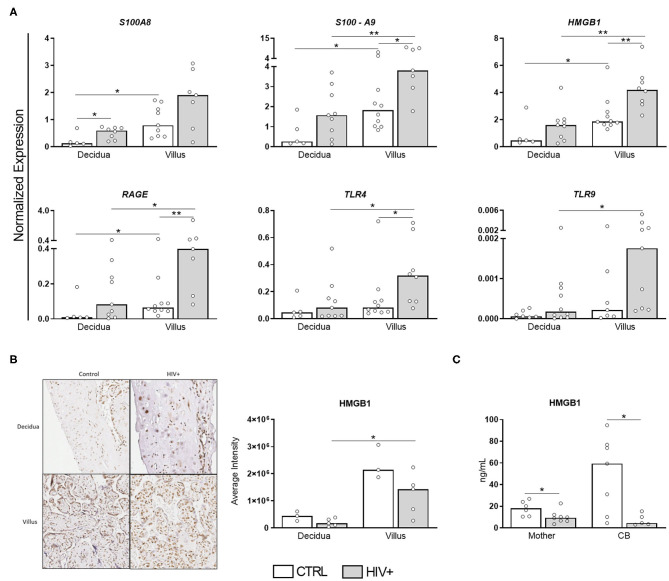
DAMPs are up-regulated in placental chorionic villi but HMGB1 serum levels are decreased in HIV-infected mothers and CB. **(A)** mRNA expression levels of S100A8, S100A9, HMGB1, RAGE, TLR4, and TLR9 in decidua and chorionic villi from HIV-infected (gray bar, *n* = 7–10) and uninfected mothers (white bar, *n* = 5–10) was evaluated by RT qPCR. **(B)** HMGB1 expression was analyzed by immunohistochemistry in decidua and villi from HIV-infected (gray bar, *n* = 5) and uninfected mothers (white bar, *n* = 3). **(C)** HMGB1 levels in serum from HIV-infected (gray bar, *n* = 5–8) and uninfected mothers/newborns pair (white bar, *n* = 6–7) evaluated by ELISA. The detection limit for HMGB1 was 2.5 ng/mL. Data represent median values. Mann-Whitney *U* or Wilcoxon signed-rank test: *p < 0.05, **p < 0.01.

A residual chronic immune activation persists in HIV-infected patients even when viral replication is efficiently inhibited by ART (17). In pregnancy, however, a tolerogenic profile, favored to promote immune tolerance toward the developing fetus, could theoretically counterbalance the HIV-associated immune activation. To assess which scenario would prevail, we evaluated the transcriptional profile of DAMPs S100A8, S100A9, and HMGB1 and their receptors RAGE, TLR4, and TLR9 in HIV-infected placentas (Figure 1).

Except for *S100A8*, all selected targets were induced in chorionic villi of HIV-infected mothers compared to control counterparts (Figure 1A). Curiously, *HMGB1* up-regulation did not translate into an enhanced protein expression in the placental tissue (Figure 1B), probably due to posttranscriptional regulation. Even more interesting, HMGB1 serum levels were remarkably reduced in HIV-infected mothers/exposed newborns. Thus, despite the up-regulated expression of DAMPs in the placenta, HIV-infected mothers do not seem to present a heightened alarmin profile systemically, suggesting pregnancy could dampen HIV-immune activation, while preserving the local response.

### Expression of Inflammasome Components in Placental Tissues From HIV-Infected Mothers

HMGB1 release is dependent on inflammasome assembly (18), but alarmins can also regulate inflammasome expression (19). Next, we analyzed the expression profile of some inflammasome components in HIV-infected mothers.

**Figure 2:**
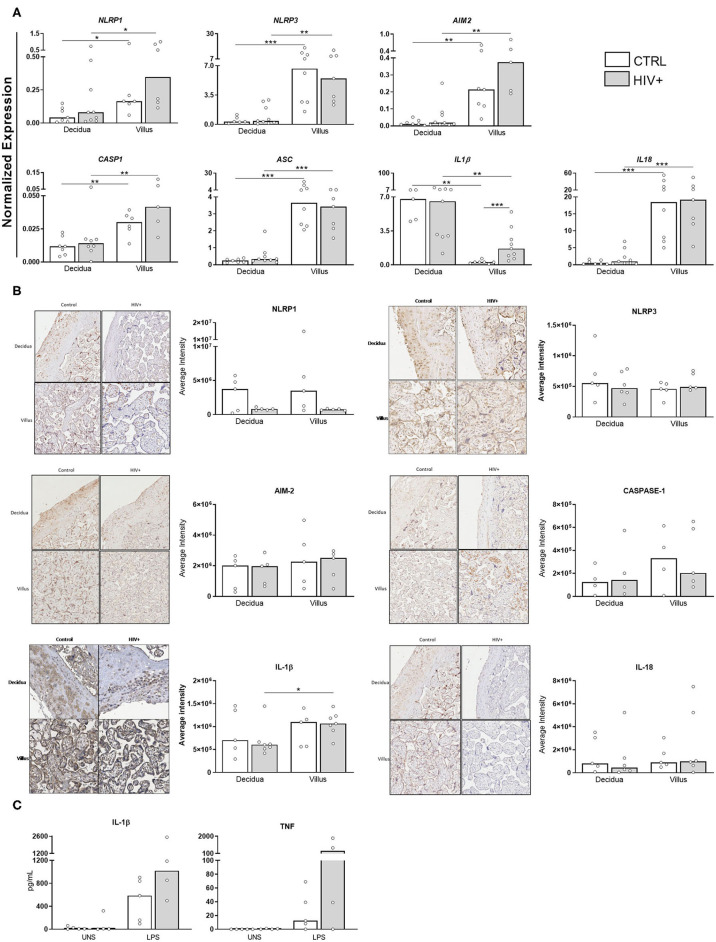
Differential expression of *IL1B* transcripts in placental chorionic villi. **(A)** mRNA expression levels of NLRP1, NLRP3, AIM-2, Caspase1, ASC, Pro-IL-1β, and Pro-IL-18 in decidua and placental chorionic villi from HIV-infected mothers (gray bar, *n* = 5–9) and control uninfected mothers (white bar, *n* = 5–8) was evaluated by RT qPCR. **(B)** NLRP1, NLRP3, AIM-2, caspase-1, IL-18, and IL-1β protein levels were analyzed by immunohistochemistry in decidua and placental chorionic villi from HIV-infected mothers (gray bar, *n* = 4–6) and uninfected mothers (white bar, *n* = 4–5). **(C)** Secretion levels of IL-1β and TNF in supernatants from placental explants from HIV-infected (gray bar, *n* = 4) and control uninfected mothers (white bar, *n* = 5) stimulated with TL4 agonist was evaluated by cytometric bead array. UNS = unstimulated. The detection limits were: IL-1β = 2.3 fg/mL; TNF = 0.7 pg/mL. Expression values are represented as median. Mann-Whitney *U* or Wilcoxon signed-rank test: *p < 0.05, **p < 0.01, ***p < 0.001.

As shown in Figure 2A, *NLRP1, NLRP3, AIM2, ASC, CASP1*, and *IL18* were preferentially expressed in villi, independent of HIV status. Intriguingly, only *IL1B* expression was higher in deciduae, and it was also enhanced in infected villi compared to control group. As verified in immunohistochemistry analyses for DAMPs, however, no differences in NLRP1, NLRP3, AIM-2, caspase-1, IL-18, and IL-1β protein expression were observed in those tissues (Figure 2B).

In addition, we analyzed the functional activity of inflammasome by measuring inflammatory cytokines in placental explants stimulated with a TLR4 agonist. Although there is no statistical difference, it is possible to observe that there is an increase in the secretion of IL-1β and TNF after agonist stimulation in both healthy and infected groups. In placentas from HIV-infected mothers, the increase of inflammatory cytokines seems to be even more intense (Figure 2C).

**Figure 3:**
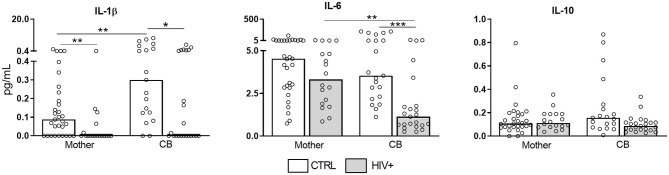
Decreased serum levels of pro-inflammatory cytokines in HIV-infected mothers and exposed newborns. Serum samples from HIV-infected mothers and cord blood (CB) (gray bar, *n* = 18–23) and control uninfected mothers and CB (white bar, *n* = 18–29) was assessed for presence of IL-1β, IL-6, and IL-10 by cytometric bead array. Data represent median values. The detection limits were: IL-10 = 13.7 fg/mL; IL-6 = 68.4 fg/mL; IL-1β = 48.4 fg/mL. Mann-Whitney *U* or Wilcoxon signed-rank test: *p < 0.05, **p < 0.01, ***p < 0.001.

We also determined the presence of pro-inflammatory cytokines in serum samples (Figure 3). Strikingly, IL-1β, and IL-6 were reduced in infected/exposed counterparts. IL-10 levels were similarly detected among our groups. IL-4 levels were not detected (data not show). These findings pointed to an intense expression of inflammatory factors in villi side from placentas that do not reflect their systemic status, reinforcing our hypothesis that pregnancy counterbalances chronic immune activation.

### Upregulation of Antiviral Factors in Placental Tissue From HIV-Infected Mothers

**Figure 4:**
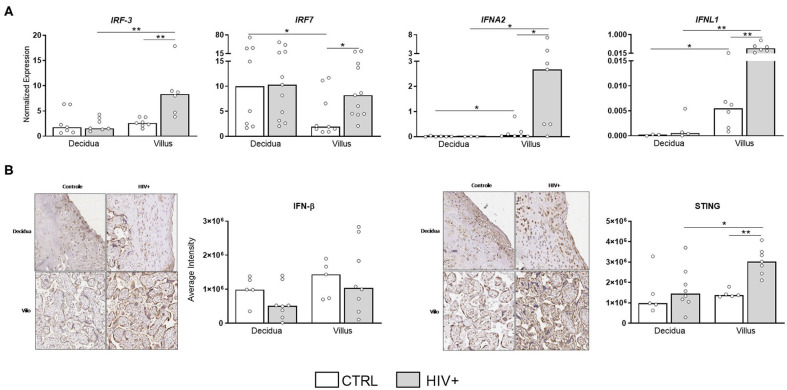
Upregulation of type I and III IFN molecules in placental tissue of HIV-infected mothers. **(A)** mRNA expression levels of IRF3, IRF7, IFNα, and IFN-λ in decidua and placental chorionic villi from HIV-infected mothers (gray bar, *n* = 6–11) and UN mothers (white bar, *n* = 3–9) were evaluated by RT qPCR. **(B)** IFN-β and STING proteins levels were analyzed by immunohistochemistry in decidua and placental chorionic villi from HIV-infected mothers (gray bar, *n* = 7–8) and UN mothers (white bar, *n* = 5). Expression levels are represented as median. Mann-Whitney *U* or Wilcoxon signed-rank test: *p < 0.05, **p < 0.01.

In addition to the observation of a pro-inflammatory status in placental villi, we also evaluated the expression of antiviral factors, particularly type I/III interferons, which could inhibit IL-1 production and inflammasome activation (20). Interestingly, type I/III IFNs, as well as their signaling molecules IRF3 and IRF7, were upregulated in placentas from HIV-infected mothers (Figure 4A), preferentially at villi compartment. STING protein expression, the main regulator of IFN production, was also increased in infected subjects, while IFN-β protein levels were equally expressed between groups (Figure 4B).

### Altered Immunological Factors Related With Tolerance in Placental Tissue From HIV-Infected Mothers

**Figure 5:**
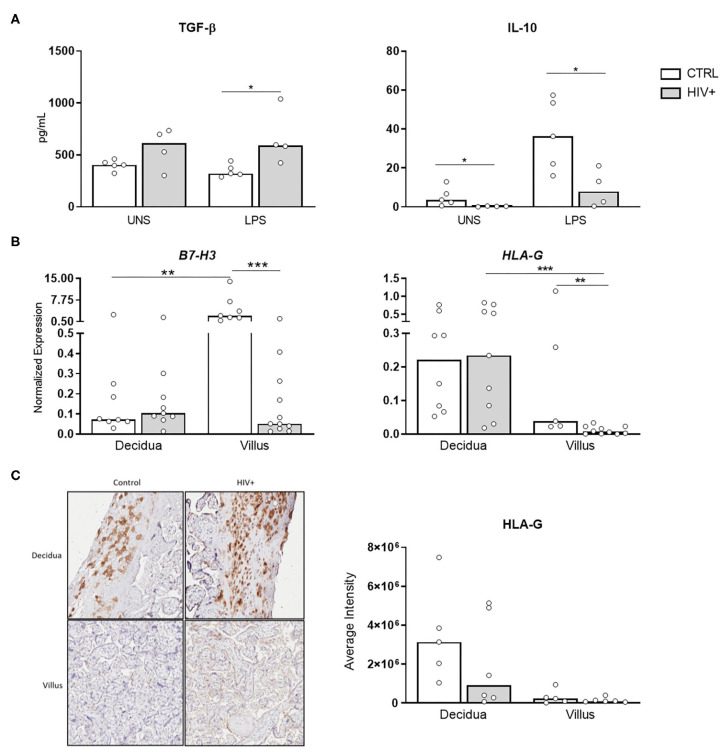
Decrease of tolerogenic factors in placental tissue from HIV-infected mothers. **(A)** Secretion levels of TGF-β and IL-10 in supernatant from placental explants from HIV-infected (gray bar, *n* = 4) and control uninfected mothers (white bar, *n* = 5) stimulated with TL4 agonist was evaluated by cytometric bead array. UNS, unstimulated. The detection limits were: IL-10, 0.13 pg/mL; TGF-β, 14.9 pg/mL. **(B)** mRNA expression levels of B7-H3 and HLA-G in decidua and placental chorionic villi from HIV-infected (gray bar, *n* = 9–11) and control uninfected mothers (white bar, *n* = 5–8) was evaluated by RT qPCR. **(C)** HLA-G protein levels were analyzed by immunohistochemistry in decidua and placental chorionic villi from HIV-infected (gray bar, *n* = 6) and uninfected mothers (white bar, *n* = 5). Expression values are represented as median. Mann-Whitney *U* or Wilcoxon signed-rank test: *p < 0.05, **p < 0.01, ***p < 0.001.

We also investigated the profile of important regulatory factors involved in maternal-fetal tolerance such as the anti-inflammatory cytokines levels, TGF-β and IL-10, in placental explants stimulated with TLR4 agonist. We observed a decrease of IL-10 secretion levels in placentas from infected mothers both constitutive and stimulated condition. In contrast, upon stimulus, increased TGF-β levels were detected in explants from HIV-infected mothers (Figure 5A).

Next, we analyzed the expression of B7-H3, a negative regulator of Th1 responses that inhibits the activity of major transcriptional factors including nuclear factor of activated T cells (21), and HLA-G. Our mRNA analysis showed that B7-H3 and HLA-G expression were reduced in villus from HIV-infected placentas compared to healthy control (Figure 5B). Moreover, HLA-G protein levels were equally expressed between the groups (Figure 5C). Transcriptional decrease of B7-H3, associated with low IL-10 levels, indicates a deficit of factors related to tolerance mechanism in HIV-infected placenta. In contrast, to rebalance this environment, HLA-G protein does not appear to be affected.

The pronounced IFN response associated with a reduction of tolerogenic factors (IL-10 and B7-H3) suggests the placenta retains the ability to induce an intense antiviral network, which could limit the fetal infection during pregnancy.

## Discussion

Innate immunity is a crucial protection mechanism in the maternal-fetal interface. In this work, we investigated the relationship between pregnancy (placenta) and three innate mechanisms important in HIV immunity. We detected an upregulation of DAMPs, inflammasome components, and type I/III IFN in placental villi from HIV-infected mothers, which did not represent the systemic profile in those women, suggesting that pregnancy could restrict the HIV-immune activation but preserve the immune response at the placental level.

The majority of our HIV-infected mothers had an undetectable viral load, and only two children became infected through vertical transmission. ART prophylaxis, recommended as a guideline from the Brazilian Ministry of Health, was applied to all infected mothers during pregnancy and delivery and to the newborn during 28 days after birth. No evidences of altered morphology were found in the placentas, probably due to their undetectable viral load levels (Table 1).

In Brazil, there is a strong adherence of HIV-infected mothers to ART, which significantly reduced vertical transmission. However, even if low, there is still a risk of infection during pregnancy or labor. In addition, the risk of viral transmission through breastfeeding ranges from 7 to 22%. It is possible that mothers whose children became infected are part of the group that has genetic/environmental characteristics that favor the infection, or that they did not strictly follow the recommendations given by doctors (postnatal treatment with AZT and not feeding newborns with breast milk). These particularities were considered in the analysis of the evaluated parameters, but no differences were found both in serum or tissue in our assays.

Regardless of the benefits of ART in HIV suppression in infected mothers, we still observed a remarkable profile of immune activation at the placenta level. Although this activation may favor fetal protection to vertical transmission, this very early stimulation may have profound impacts on the development of the immune system (22, 23), which are commonly seen in HIV-exposed newborns.

Intriguingly, we observed contradictory results between HMGB1 transcriptional and protein levels, suggestive of post-transcriptional regulation. HMGB1 can favor HIV replication (24), but regulatory microRNAs can outweigh its production to avoid local infection. Indeed, miRNA-218 and miRNA-1284 have been described for suppressing HMGB1 in lung cancer and osteosarcoma models, respectively (25, 26).

Alongside HMGB reduction, pro-inflammatory cytokines, as IL-1β, were also repressed. We ruled out that this phenomenon could be associated to ART since immune activation persists even in HIV-infected patients in which viral replication was inhibited by treatment (17), thus, the immune regulatory mechanism associated to pregnancy seems to be the principal driving force behind our results.

During acute phase of HIV infection, the increased type I IFN response induces several antiviral factors that inhibit the infection (27). However, in chronic phase, this persistent IFN production desensitizes host cells, resulting in an inappropriate antiviral response that leads to disease progression (28). In our results, we observed an increased IFN response in placental tissues. It remains to be explored in future works whether this heightened response protects the fetus at the same time it compromises its immune development.

In conclusion, our findings suggest the placenta retains its immunological potential to respond to HIV infection while the pregnancy status counterpoises the immune activation systemically. This complex regulation can both control vertical transmission and also lead to immunological dysfunction of the newborn. A deep understanding of the immunological networks in HIV infection during pregnancy may be key to uncover efficient therapeutic targets and contribute to the quality of life of those patients.

## Data Availability Statement

The datasets generated for this study are available on request to the corresponding author.

## Ethics Statement

The studies involving human participants were reviewed and approved by Institutional Use Committee of the University of São Paulo and University of Campinas (Plataforma Brasil database, CAAE: 31605314.3.0000.0068). The patients/participants provided their written informed consent to participate in this study.

## Author Contributions

NP, AB, KM, JL, FY, NP, and MSo performed experiments. NP, AB, KM, JL, FY, and HM recruited the patients. NP, AD, and MSa participated in designing research. NP and MSa wrote the paper. All authors contributed to the article and approved the submitted version.

## Conflict of Interest

The authors declare that the research was conducted in the absence of any commercial or financial relationships that could be construed as a potential conflict of interest.
